# Supplementary material for: Single-base tiled screen unveils design principles of PspCas13b for potent and off-target-free RNA silencing
Source: Nat Struct Mol Biol. 2024 Jul 1;31(11):1702–16. doi: 10.1038/s41594-024-01336-0 (PMC11564092; doi:10.1038/s41594-024-01336-0)
Supplement: Supplementary file 1 — Supplementary discussion, Fig. 1 and Tables 1, 4 and 5. [file 41594_2024_1336_MOESM1_ESM.pdf]

nature structural & molecular biology

Supplementary information https://doi.org/10.1038/s41594-024-01336-0

Single-base tiled screen unveils design principles of PspCas13b for potent and off-target-free RNA silencing

In the format provided by the authors and unedited

Table of Contents

SUPPLEMENTARY DISCUSSION: 2

Supplementary Figure 1. 4

Supplementary Table 1. Predicted probabilities of PspCas13b off-targeting other transcripts in the human transcriptome based on spacer length and mismatch tolerance thresholds. 5

Supplementary Table 4. Primer sequences used in this study 7

Supplementary Table 5. Transfection conditions of HEK 293T cells. 7

SUPPLEMENTARY DISCUSSION:

5'GG motif enhances crRNA transcription and pspCas13b catalytic activity

We found that the intracellular abundance of 5'GG crRNAs was increased, possibly due to enhanced transcription (**Extended Data Fig. 6**). This is consistent with a previous report that Pol III promoters can yield a higher transcription rate when the transcribed small RNA has a 5' A or G base[1]. Direct transfection of IVT or synthetic crRNAs showed that the G-rich motif can indeed further improve crRNA potency through other means, such as enhanced crRNA stability, loading (affinity with *Psp*Cas13b), or by conferring greater nuclease activity beyond target recognition (**Figure 4**). In addition, recombinant *Psp*Cas13b loaded with synthetic crRNAs containing a mismatched 5'GG motif exhibited higher cleavage activity *in-vitro* compared to fully basepaired crRNAs lacking a 5'GG motif.

5'GG motif increases the targeting window of PspCas13b

When seeking to silence a selected transcript, there are usually numerous options for crRNA design, allowing the selection of a crRNA that intrinsically commences with a 5'GG and can therefore fully basepair with the target. Conversely, when the target window is restricted to a specific location on a transcript (e.g., targeting a specific RNA isoform, single-nucleotide variants, or the breakpoint of gene fusions), the options for crRNA design with a natural 5'GG motif are more limited. In such instances, introducing a non-cognate 5'GG can augment the silencing efficiency of an otherwise ineffective crRNA.

Lack of "Seed"

In many RNA-guided nucleases like Cas9, Cas12, and Ago2, mismatch intolerance can be determined by surface-exposed mismatch-sensitive 'pre-seed' or 'seed' regions within their guide RNA. These microfeatures facilitate efficient target search and recognition processes through the nucleation of RNA-DNA or RNA-RNA basepairing[2,3]. Although we observed some levels of variation in mismatch intolerance at various regions, our mutagenesis data did not reveal any unique 'pre-seed' or 'seed'-like region within the spacer of *Psp*Cas13b that is highly sensitive to single, double, or triple nucleotide mismatches beyond the 5'GG motif we discussed above (**Figure 6**).

Mismatch tolerance is dependent on spacer length

The comparative mutagenesis study also indicated that the mismatch tolerance threshold is dependent on the length of the spacer. Cas13 orthologs with longer spacer sequences are more likely to accommodate a higher number of unpaired nucleotides (**Extended Data Fig. 8**). Notably, *Psp*Cas13b displayed a slightly higher mismatch tolerance than *Rfx*Cas13d, which is likely attributable to the disparities in their spacer length. Accordingly, when the spacer length of *Psp*Cas13b was shortened from 30 to 27 nucleotides, its mismatch tolerance was reduced (**Extended Data Fig. 8**).

Mismatch tolerance and intolerance

Our comprehensive mutagenesis study revealed the interface between mismatch tolerance and intolerance (Figure 6, Extended Data Figure 8). Leveraging this

transition from mismatch tolerance to intolerance may enable selective silencing of RNA isoforms that share extensive sequence homology.

PspCas13b is highly specific

Our proteomic data demonstrated that PspCas13b is highly specific and lacks any collateral activity. Mechanistically, the lack of collateral activity is perhaps attributable to the unique domain rearrangement of the Cas13b family which is distinct from other type VI CRISPR effectors. Although the HEPN-1 and HEPN-2 domains are located at the extreme N and C protein termini, upon protein folding the two RXXXXH motifs become relatively juxtaposed[4–6]. This structural singularity may limit the surface-exposure of *PspCas13b* activated HEPN nuclease domains, enabling it to retain high on-target silencing activity without exhibiting significant collateral activity in human HEK 293T cells.

It is important to note that the collateral activity of other Cas13 enzymes in mammalian cells remains controversial. The controversy in the literature may be attributed to inadequate controls and the absence of rigorous orthogonal approaches to precisely assess the extent of collateral activity on endogenous transcripts and proteins. For example, some earlier studies relied on fluorescence assays as a surrogate to gauge Cas13 collateral activity against non-target, overexpressed reporter transcripts. However, these assays may not fully capture the genuine impact of collateral activity on the endogenous human proteome. Therefore, we suggest that a systematic proteomic analysis of various Cas13 enzymes' could provide a more comprehensive understanding of their specificity and the scope of collateral activity in mammalian cells.

Limitations of the study.

In this proof-of-concept study, we investigated the molecular basis of PspCas13b and its specificity in HEK 293T human cell line. Future proteomic analyses in other eukaryotic cell lines, primary cells, and animal models are required for a deeper understanding of the on-target and collateral activity of various Cas13 orthologs.

Supplementary Figure 1

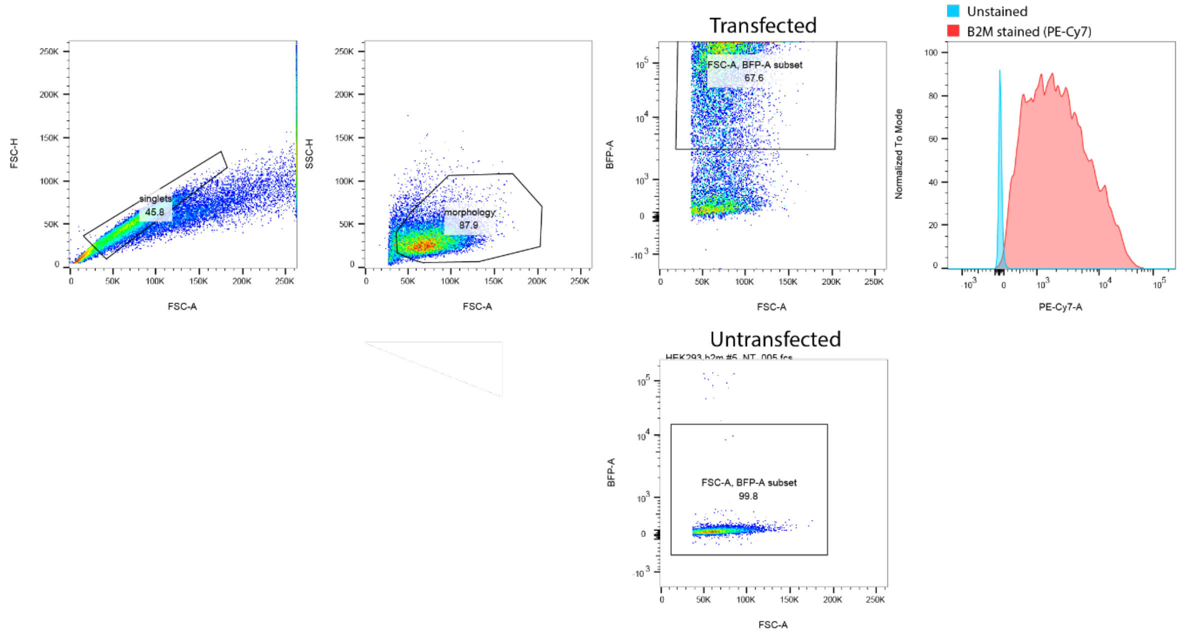

Supplementary Figure 1. FACS gating strategy used to analyze the expression level of the surface marker B2M in HEK293 T cells transfected with *Psp*Cas13b and various crRNAs. We gated on singlets to exclude cell aggregations, followed by gating on HEK293 T cell morphology, and gating on PspCas13b-BFP^{+} positive cell population. In the BFP positive population, the expression level of B2M was further analyzed. A representative B2M FACS histogram obtained with this gating strategy is displayed in the histogram (top right panel) comparing unstained and B2M strained cells.

Supplementary Table 1. Predicted probabilities of PspCas13b off-targeting other transcripts in the human transcriptome based on spacer length and mismatch tolerance thresholds.

Minimum basepairing required for *Psp*Cas13b activation (mismatch tolerance threshold)	Probability of off-targeting other RNA in the human protein coding transcriptome (size ~$6x10^7$-nt)	Probability of off-targeting other RNA in the human transcriptome (coding & non-coding) (size ~$2.3x10^9$-nt)
26-nt basepairing required<br>If four mismatches tolerance)	***$P=(6x10^7)/(4^{26}) =1.332x10^{-8}$***<br>(Chance of occurrence: 0.000001332%)	***$P=(2.3x10^9)/(4^{26})=5.107x10^{-7}$***<br>(Chance of occurrence: 0.00005107%)
25-nt basepairing required<br>(Five mismatches tolerance)	***$P=(6x10^7)/(4^{25}) =5.329 x10^{-8}$***<br>(Chance of occurrence: 0.000005329%)	***$P=(2.3x10^9)/(4^{25})=2.042x10^{-6}$***<br>(Chance of occurrence: 0.0002042%)
24-nt basepairing required<br>(Six mismatches tolerance)	***$P=(6x10^7)/(4^{24}) =2.131x10^{-7}$***<br>(Chance of occurrence: 0.00002131%)	***$P=(2.3x10^9)/(4^{24})=8.171x10^{-6}$***<br>(Chance of occurrence: 0.0008171%)

Note: Our comprehensive mutagenesis study revealed that *Psp*Cas13b can tolerate around three to four nucleotide mismatches with the target, and beyond this mismatch threshold, the catalytic activity becomes largely impaired (**Figure 6**). Based on this targeting resolution and assuming a uniform distribution of nucleotides, we were able to predict the potential for off-targeting other cellular transcripts in the human transcriptome. This was based on the probability of encounter with cellular transcripts that would have 26, 25, or 24 nucleotide basepairing with a given spacer sequence (**Suppl. Table 1**).

Probabilities of off-targeting RNA in the human protein coding transcriptome: The size of human protein coding transcriptome is approximatively $6x10^7$ nucleotides.

- If *Psp*Cas13b tolerates four nucleotides mismatch with the target, the probability of finding a 26-nucleotide long sequence that could basepair with the spacer is $(1/4)^{26}$. With this mismatch threshold, the chance of off-targeting other RNA in the human protein coding transcriptome is 0.000001332% *($P=6x10^7/4^{26} =1.332x10^{-8}$).*
- If *Psp*Cas13b tolerates five nucleotides mismatch with the target, the probability of finding a 25-nucleotide long sequence that could basepair with the spacer is $(1/4)^{25}$. With this mismatch threshold, the chance of off-targeting other RNA in the human protein coding transcriptome is 0.000005329% *($P=6x10^7/4^{25} =5.329 x10^{-8}$).*
- If *Psp*Cas13b tolerates six nucleotides mismatch with the target, the probability of finding a 24-nucleotide long sequence that could basepair with the spacer is $(1/4)^{24}$. With this mismatch threshold the chance of off-targeting other RNA in the human protein coding transcriptome is 0.00002131% *($P=6x10^7/4^{24} =2.131x10^{-7}$).*

Probability of off-targeting RNA in the human coding and non-coding transcriptome: The size of human coding and non-coding transcriptome is approximatively $2.3x10^9$ nucleotides.

- If *Psp*Cas13b tolerates four nucleotides mismatch with the target, the probability of finding a 26-nucleotide long sequence that could basepair with the spacer is $(1/4)^{26}$. With this mismatch threshold, the chance of off-targeting other RNA in the human coding and non-coding transcriptome is 0.00005107% *($P=2.3x10^9/4^{26}=5.107x10^{-7}$).*
- If *Psp*Cas13b tolerates five nucleotides mismatch with the target, the probability of finding a 25-nucleotide long sequence that could basepair with the spacer is $(1/4)^{25}$. With this mismatch threshold, the chance of off-targeting other RNA in the human coding and non-coding transcriptome is 0.0002042% *($P=2.3x10^9/4^{25}=2.042x10^{-6}$).*
- If *Psp*Cas13b tolerates six nucleotides mismatch with the target, the probability of finding a 24-nucleotide long sequence that could basepair with the spacer is $(1/4)^{24}$. With this

mismatch threshold the chance of off-targeting other RNA in the human coding and non-coding transcriptome is 0.0008171% *($P=2.3x10^9/4^{24}=8.171x10^{-6}$).*

These estimations of the probability of occurrence of off-targeting transcriptome-wide suggest that *Psp*Cas13b should maintain an extremely high specificity thanks to its long spacer sequence and low mismatch tolerance threshold. This prediction of the high specificity of *Psp*Cas13b is confirmed by mass spectrometry analysis (**Figure 7**).

Supplementary Table 4. Primer sequences used in this study

Primer name	Sequence	Description
pLXSN 5' forward	CCCTTGAACCTCCTCGTTCGACC	Sanger sequence primer for BCR-ABL1/BCR/ABL1 fragment cloning
M13 reverse	CAGGAAACAGCTATGAC	Sanger sequence primer for crRNA cloning
crRNA DR RT-PCR reverse	GTTGTAATAGCCCCTCAAAAC	RT-PCR for crRNA level
crRNA mCherry tiled 39/40 RT-PCR forward	GTACATCCGCTCGGAGGA	RT-PCR for crRNA level
crRNA mCherry RT-PCR tiled 26/27 forward	CCGTCCTCGGGGTACATC	RT-PCR for crRNA level
HSP90A1B RT-PCR forward	AGAAATTGCCCAACTCATGTCC	RT-PCR for HSP90A1B mRNA level (housekeeping)
HSP90A1B RT-PCR reverse	ATCAACTCCCGAAGGAAAATCTC	RT-PCR for HSP90A1B mRNA level (housekeeping)
GAPDH RT-PCR forward	GGAGCGAGATCCCTCCAAAAT	RT-PCR for GAPDH mRNA level (housekeeping)
GAPDH RT-PCR reverse	GGCTGTTGTCATACTTCTCATGG	RT-PCR for GAPDH mRNA level (housekeeping)
5S ribosomal RNA forward	CGTCTGATCTCGGAAGCTAAG	RT-PCR for 5s rRNA level (housekeeping)
5S ribosomal RNA Reverse	CCTACAGCACCCGGTATTC	RT-PCR for 5s rRNA level (housekeeping)

Supplementary Table 5. Transfection conditions of HEK 293T cells.

Component (per well)	96-well	24-well	12-well
# of seeded HEK 293T cells	30,000	150,000	300,000
Plasmids DNA amount	100ng	500ng	1000 ng
P3000 reagent	0.2μL	1μL	2 μl
Lipofectamine 3000 reagent	0.3μL	1.5μL	3 μl
Opti-MEM	Up to 10 μL	Up to 50 μL	Up to 100 μL

REFERENCES

1. Ma, H. *et al.* Pol III promoters to express small RNAs: Delineation of transcription initiation. *Mol. Ther. - Nucleic Acids* **3**, (2014).
2. Stella, S. *et al.* Conformational Activation Promotes CRISPR-Cas12a Catalysis and Resetting of the Endonuclease Activity. *Cell* **175**, (2018).
3. Chandradoss, S. D., Schirle, N. T., Szczepaniak, M., Macrae, I. J. & Joo, C. A Dynamic Search Process Underlies MicroRNA Targeting. *Cell* **162**, (2015).
4. Slaymaker, I. M. *et al.* High-Resolution Structure of Cas13b and Biochemical Characterization of RNA Targeting and Cleavage. *Cell Rep.* **26**, (2019).
5. Shmakov, S. *et al.* Diversity and evolution of class 2 CRISPR-Cas systems. *Nat. Rev. Microbiol.* **15**, (2017).
6. Zhang, B. *et al.* Structural insights into Cas13b-guided CRISPR RNA maturation and recognition. *Cell Research* vol. 28 (2018).
