# Supplementary material for: Single-base tiled screen unveils design principles of PspCas13b for potent and off-target-free RNA silencing
Source: Nat Struct Mol Biol. 2024 Jul 1;31(11):1702–16. doi: 10.1038/s41594-024-01336-0 (PMC11564092; doi:10.1038/s41594-024-01336-0)
Supplement: Supplementary file 2 — Reporting Summary [file 41594_2024_1336_MOESM2_ESM.pdf]

nature portfolio

Corresponding author(s): Mohamed Fareh, PhD

Last updated by author(s): Mar 14, 2024

Reporting Summary

Nature Portfolio wishes to improve the reproducibility of the work that we publish. This form provides structure for consistency and transparency in reporting. For further information on Nature Portfolio policies, see our Editorial Policies and the Editorial Policy Checklist.

Statistics

For all statistical analyses, confirm that the following items are present in the figure legend, table legend, main text, or Methods section.

n/a	Confirmed	
☐	☒	The exact sample size (*n*) for each experimental group/condition, given as a discrete number and unit of measurement
☐	☒	A statement on whether measurements were taken from distinct samples or whether the same sample was measured repeatedly
☐	☒	The statistical test(s) used AND whether they are one- or two-sided<br>*Only common tests should be described solely by name; describe more complex techniques in the Methods section.*
☒	☐	A description of all covariates tested
☒	☐	A description of any assumptions or corrections, such as tests of normality and adjustment for multiple comparisons
☐	☒	A full description of the statistical parameters including central tendency (e.g. means) or other basic estimates (e.g. regression coefficient) AND variation (e.g. standard deviation) or associated estimates of uncertainty (e.g. confidence intervals)
☐	☒	For null hypothesis testing, the test statistic (e.g. *F*, *t*, *r*) with confidence intervals, effect sizes, degrees of freedom and *P* value noted<br>*Give P values as exact values whenever suitable.*
☒	☐	For Bayesian analysis, information on the choice of priors and Markov chain Monte Carlo settings
☒	☐	For hierarchical and complex designs, identification of the appropriate level for tests and full reporting of outcomes
☒	☐	Estimates of effect sizes (e.g. Cohen's *d*, Pearson's *r*), indicating how they were calculated

Our web collection on statistics for biologists contains articles on many of the points above.

Software and code

Policy information about availability of computer code

Data collection

The bioinformatic codes for the design of predicted potent crRNA is available at https://github.com/david-ma/cas13. crRNA design web-server described in this study is available at: https://cas13target.azurewebsites.net/.

Data analysis

The design of in silico prediction tool is based on design principles learned from the experimental data presented in this article. The R (version 4.2.2) programming language was used for coding and the R Shiny framework (version 1.7.4) was used to develop the software application. The software program is deployed as a web application using the Microsoft Azure platform. Briefly, the software takes an RNA or DNA sequence and generates all single-nucleotide tiled spacers using the input sequence. The program then removes all spacer sequences that possess more than three consecutive T bases (>3T) that are predicted to act as a transcription termination signal and could yield premature crRNAs. The algorithm scores the remaining spacer sequences based on their nucleotide composition and position. Spacers with a G nucleotide at the first or second positions receive a maximum score of +60 each. In contrast, a C nucleotide at spacer position 1, 2, 3, or 4 receives a penalty score of -60, -60, -50, and -40 each, respectively. Additionally, C bases at positions 11, 12, 15, 16, and 17 receive a -5 score each. All other nucleotides or spacer positions that did not show any enrichment in the potent and ineffective crRNA cohorts receive a score of 0. The algorithm then calculates the cumulative score for each spacer and ranks them accordingly. As a result, the top spacers with high scores are enriched with G bases at 1st and 2nd positions, and depleted from C bases at positions 1, 2, 3, 4, 11, 12, 15, 16, and 17, and are predicted to yield potent silencing. Conversely, the lowest scoring spacers at the bottom of the list are enriched with C bases at positions 1, 2, 3, 4, 11, 12, 15, 16, and 17 and are predicted to yield ineffective silencing. The prediction accuracy of the algorithm is supported by in silico analysis and functional validation data in Figures 3, 4, and 5 and Supplementary Figures 3, 4, 5 & 6. In addition to identifying potent crRNAs for PspCas13b, the web application also assesses potential off-target effects of the top 10 predicted potent spacer sequences based on their sequence complementarity with various RNA transcripts in the human transcriptome. The web application integrates the NCBI BLAST (Basic Local Alignment Search Tool) command line tool for this purpose and reports the on-target and off-target(s) within the human transcriptome (GRCh38.p14; Annotation Name: GCF_000001405.40-RS_2023_10 (October 2, 2023)). The webpage displays the percentage of match and

number of nucleotide mismatches with other RNA molecules that possess sequence complementarity with the selected spacer sequence. The software categorizes off-target effects as nonexistent when the number of mismatches is greater than 15. Building on mutagenesis studies, we predict that crRNAs with partial sequence complementarity, involving 6 nucleotide mismatches or longer, are likely to lose their silencing activity. Consequently, any off-target transcript displayed on the prediction webpage with 6 or more mismatches is considered unlikely to be silenced. In cases where potential off-targets are identified, the output provides a link to the NCBI records of these human transcripts. To expedite the processing of this web application, we recommend utilizing DNA or RNA coding sequences (CDS) with a length shorter than 1000 nucleotides as input.
This PspCas13b crRNA design tool developed in this study (version 1) is open source and available to the wider scientific community at https://cas13target.azurewebsites.net (Extended Figure 10).

The predicted RNA secondary structures and minimum free energy were generated using the RNAfold program (ViennaRNA webservices; Lorenz, R. et al. ViennaRNA Package 2.0. Algorithms Mol. Biol. (2011) doi:10.1186/1748-7188-6-26.).

RNA hybridization/interaction energy, crRNA spacer GC content, and A/U/G/C content. The R package 'ggseqlogo' was used to assess nucleotide preference in crRNA spacer and PFS sequence. Delta probability graphs of spacer nucleotides were generated with Matplotlib.

Data analyses and visualizations (graphs) were performed in GraphPad Prism software version 9, unless stated otherwise.

For manuscripts utilizing custom algorithms or software that are central to the research but not yet described in published literature, software must be made available to editors and reviewers. We strongly encourage code deposition in a community repository (e.g. GitHub). See the Nature Portfolio guidelines for submitting code & software for further information.

Data

Policy information about availability of data

All manuscripts must include a data availability statement. This statement should provide the following information, where applicable:

- Accession codes, unique identifiers, or web links for publicly available datasets
- A description of any restrictions on data availability
- For clinical datasets or third party data, please ensure that the statement adheres to our policy

All the raw data supporting the findings are available in the source Data file submitted with this manuscript.
All data are available in the main text and supplementary materials. Source Data are available on Figshare (10.6084/m9.figshare.25058588). All key plasmids constructed in this study, their sequences, and maps will be deposited to Addgene upon publication.
Code Availability. The bioinformatic codes for the design of predicted potent crRNA is available at https://github.com/faraz107/cas13target. crRNA design webserver described here is available at: https://cas13target.azurewebsites.net/.
Human transcriptome (GRCh38.p14; Annotation Name: GCF_000001405.40-RS_2023_10 (October 2, 2023)) and Human reference proteomes (UP000005640) are used in this study

Research involving human participants, their data, or biological material

Policy information about studies with human participants or human data. See also policy information about sex, gender (identity/presentation), and sexual orientation and race, ethnicity and racism.

Reporting on sex and gender	N/A
Reporting on race, ethnicity, or other socially relevant groupings	N/A
Population characteristics	N/A
Recruitment	N/A
Ethics oversight	N/A

Note that full information on the approval of the study protocol must also be provided in the manuscript.

Field-specific reporting

Please select the one below that is the best fit for your research. If you are not sure, read the appropriate sections before making your selection.

☒ Life sciences ☐ Behavioural & social sciences ☐ Ecological, evolutionary & environmental sciences

For a reference copy of the document with all sections, see nature.com/documents/nr-reporting-summary-flat.pdf

Life sciences study design

All studies must disclose on these points even when the disclosure is negative.

Sample size	The sample sizes were determined to match the standards in comparable studies available in the literature (Chunlong Xu et at, Nat Methods,

Sample size	2021).
Data exclusions	Experiments and protocols were optimized in pilot assays before generating high-quality publication data. No data was excluded from the analysis.
Replication	All experiments were repeated at least 3 times as biological replicates with the following exceptions:<br>- As mentioned in the figure legend, Data in Fig 2b (screening 61 single-base tiled crRNAs targeting mCherry mRNA) was performed in two biological replicates due to the large size of crRNAs screened.<br>After the initial optimization of the experimental conditions, all experiments were reproducible in independent experiments. RNA targeting with CRISPR-Cas13 is well-established and similar silencing experiments using various Cas13 tools have been reported by independent researchers.
Randomization	No randomization was used in this study. Due to the small sample, randomization was not relevant for this study. Covariates were controlled for by running controls in parallel whenever is applicable. Appropriate controls (e.g. non targeting crRNAs, dpspCas13b, crRNA alone, loading controls in WB, and crRNA dose-dependent silencing) were used throughout the study.
Blinding	No blinding was used in this study. Blinding is not relevant to this study as RNA targeting with Cas13 is well-established in the field by independent groups using assays that do not require blinding (Chunlong Xu et at, Nat Methods, 2021). Most experiments were performed, analysed, and confirmed by independent co-authors in our labs.

Reporting for specific materials, systems and methods

We require information from authors about some types of materials, experimental systems and methods used in many studies. Here, indicate whether each material, system or method listed is relevant to your study. If you are not sure if a list item applies to your research, read the appropriate section before selecting a response.

Materials & experimental systems

n/a	Involved in the study
☐	☒ Antibodies
☐	☒ Eukaryotic cell lines
☒	☐ Palaeontology and archaeology
☒	☐ Animals and other organisms
☒	☐ Clinical data
☒	☐ Dual use research of concern
☒	☐ Plants

Methods

n/a	Involved in the study
☒	☐ ChIP-seq
☐	☒ Flow cytometry
☒	☐ MRI-based neuroimaging

Antibodies

Antibodies used	Antibidies used in this study are listed below:<br>- Monoclonal β actin antibody (AC-74) (source: mouse, western blot application: 1:2000) Sigma-Aldrich A2228<br>- Monoclonal ANTI-FLAG antibody (M2) (source: mouse, western blot application: 1:2000 Sigma-Aldrich F1804<br>-Monoclonal ANTI-HA-Tag (6E2) antibody (source: mouse, western blot application: 1:1000) Cell Signalling Technology 2367<br>-Monoclonal ANTI-mCherry (E5D8F) antibody (source: rabbit , western blot application: 1:1000) Cell Signalling Technology 43590<br>- Polyclonal c-ABL antibody (source: rabbit, western blot application: 1:2000) Cell Signaling Technology 2862<br>- Monoclonal PE/Cyanine7 anti-human β2-microglobulin Antibody (2M2) (FACS application: 1:500) BioLegend 316317<br>- Polyclonal (Horseradish peroxidase) HRP conjugated goat anti-mouse IgG secondary Antibody (western blot application: 1:10,000) Abcam ab97023<br>- Polyclonal HRP conjugated goat anti-rabbit IgG secondary Antibody (western blot application: 1:2000) Abcam ab205718
Validation	We used commercial antibodies validated by the suppliers. We confirmed the validation as we used unstained cells, untransfected cells, a secondary-antibody only control, and other appropriate controls to validate the specificity of various antibodies we used in this study.<br>Monoclonal Anti-β-Actin antibody has been used in western blot and two-dimensional gel immunoblot.<br>Anti-flag AB: For highly sensitive and specific detection of FLAG fusion proteins by immunoblotting, immunoprecipitation (IP), immunohistochemisty, immunofluorescence and immunocyotchemistry. Optimized for single banded detection of FLAG fusion proteins in mammalian, plant, and bacterial expression systems.<br>HA-Tag (6E2) Mouse mAb detects recombinant proteins containing the HA epitope tag. The antibody recognizes the HA-tag fused to either the amino or carboxy terminus of targeted proteins in transfected cells.<br>mCherry (E5D8F) Rabbit mAb detects mCherry-tagged proteins (either N-terminal tagged or C-terminal tagged) exogenously expressed in cells. Please note that the mCherry tags add approximately 28kDa to the molecular weight of the fusion protein.<br>Polyclonal antibodies are produced by immunizing animals with a synthetic peptide corresponding to residues surrounding Pro580 of human c-Abl. Antibodies are purified by protein A and peptide affinity chromatography.<br>Monoclonal PE/Cyanine7 anti-human β2-microglobulin Antibody: The antibody was purified by affinity chromatography and conjugated with PE/Cyanine7 under optimal conditions.

Eukaryotic cell lines

Policy information about cell lines and Sex and Gender in Research

Cell line source(s)	HEK 293T (ATCC CRL-3216)
Authentication	Cell lines were authenticated by the supplier ATCC. We did not perform any additional authentication upon reception. We made a bulk stocks for each cell line after recovering from the original frozen vials. We discard the cells after ~20 passages, and thaw new cells from the liquid nitrogen stocks. Cell morphology was monitored at each passage by microscope.
Mycoplasma contamination	Cells were monthly tested for mycoplasma contamination (QPCR based test and microscopy) and were mycoplasma negative.
Commonly misidentified lines (See ICLAC register)	No commonly misidentified cell lines were used in this manuscript.

Flow Cytometry

Plots

Confirm that:

☒ The axis labels state the marker and fluorochrome used (e.g. CD4-FITC).

☒ The axis scales are clearly visible. Include numbers along axes only for bottom left plot of group (a 'group' is an analysis of identical markers).

☒ All plots are contour plots with outliers or pseudocolor plots.

☒ A numerical value for number of cells or percentage (with statistics) is provided.

Methodology

Sample preparation	For B2M surface marker staining, up to 1x106 cells were incubated in 50 µL of PBS/2% FBS (v/v) containing B2M antibody for 30 minutes on ice in the dark. The cells were then washed twice with 200 µL of PBS/2% (v/v) FBS before being re-suspended in 200 µL of PBS/2% FBS (v/v) for flow cytometry analysis.<br>Supplementary Table 5 lists the antibodies that were used in this study including for FACS analysis.<br>- Monoclonal PE/Cyanine7 anti-human β2-microglobulin Antibody (2M2) (FACS application: 1:500) BioLegend 316317
Instrument	Flow cytometry analysis was performed using either the FACS Symphony Cell Analyzer A5 or A3 (BD Biosciences).
Software	All flow cytometry profiles were analyzed using FlowJo V10 software (Tree Star Inc).
Cell population abundance	For antibody-stained samples, the cell purify was compared to a unstained control, a secondary-antibody only control or a IgG isotype control.
Gating strategy	The gating strategies are detailed in the Supplementary Fig. 1

☒ Tick this box to confirm that a figure exemplifying the gating strategy is provided in the Supplementary Information.
