# Supplementary figures and images for: Single-base tiled screen unveils design principles of PspCas13b for potent and off-target-free RNA silencing
Source: Nat Struct Mol Biol. 2024 Jul 1;31(11):1702–16. doi: 10.1038/s41594-024-01336-0 (PMC11564092; doi:10.1038/s41594-024-01336-0)

Extended.Fig.1d

NT

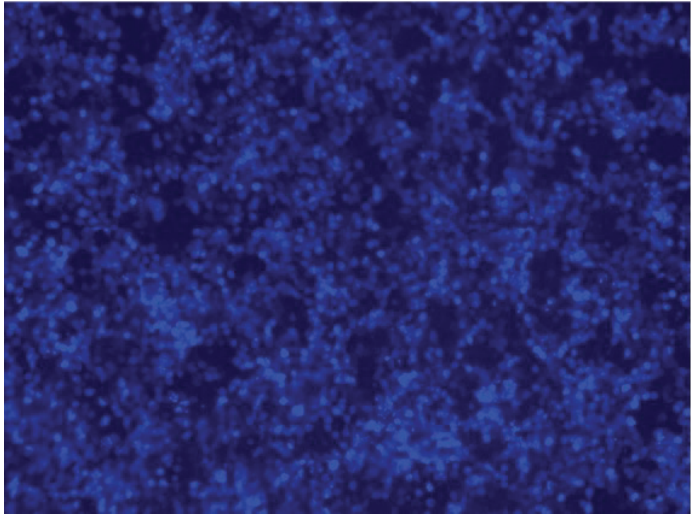

crRNA12

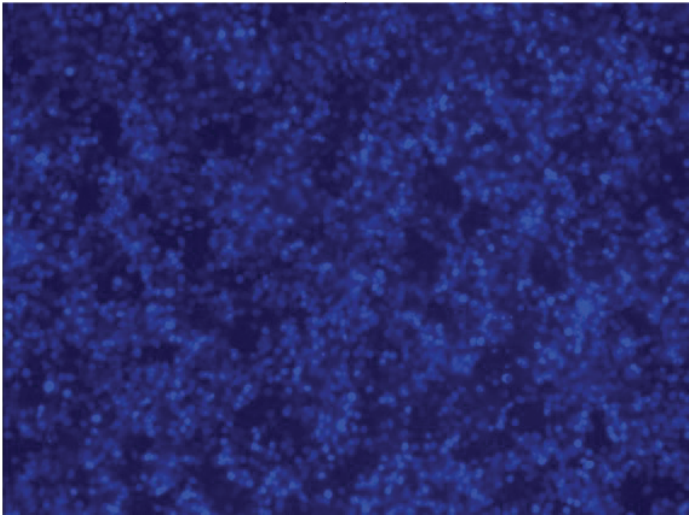

Supplement: Supplementary file 5 — Source data for Extended Data Fig. 1. [file 41594_2024_1336_MOESM5_ESM.pdf]

Extended. Fig. 2b

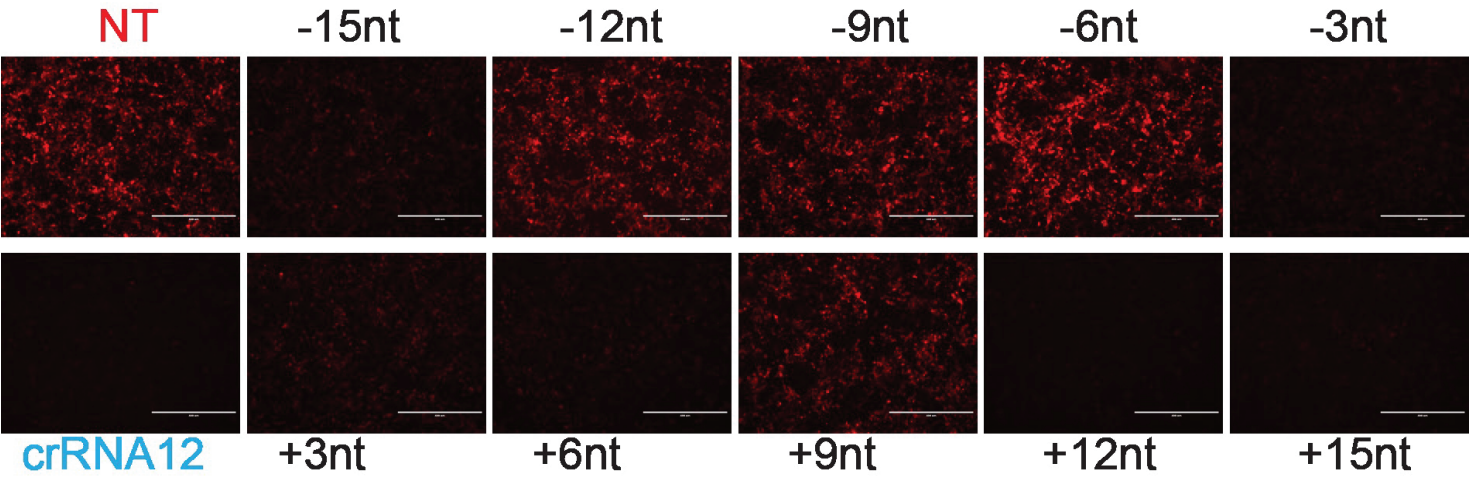

Extended. Fig. 2b

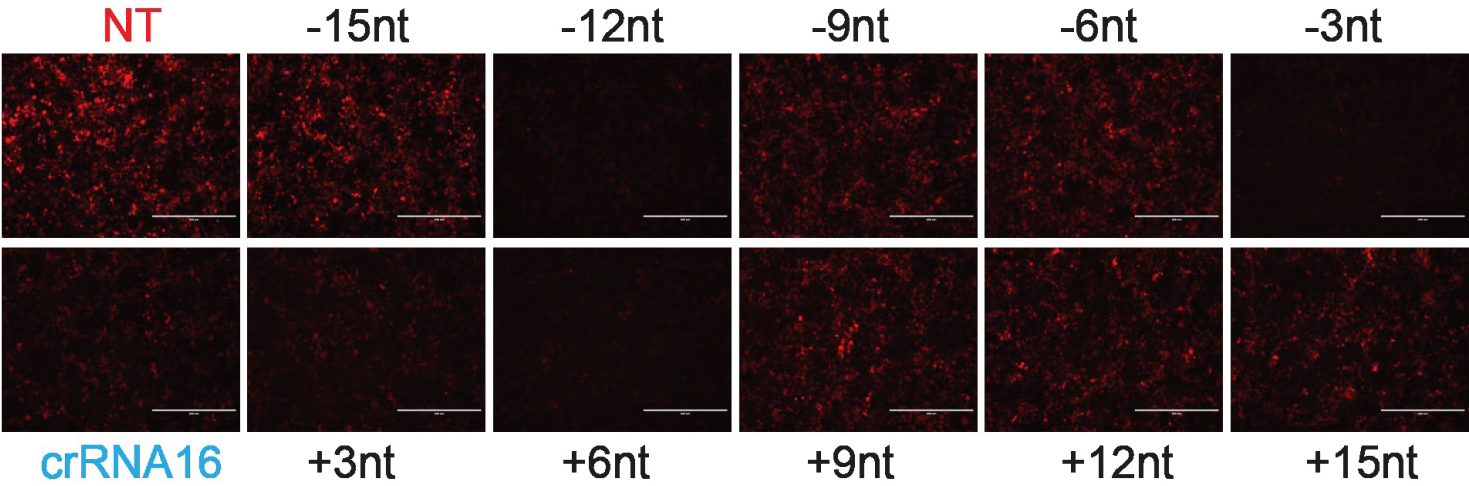

Supplement: Supplementary file 6 — Source data for Extended Data Fig. 2. [file 41594_2024_1336_MOESM6_ESM.pdf]

Extended. Fig. 5c

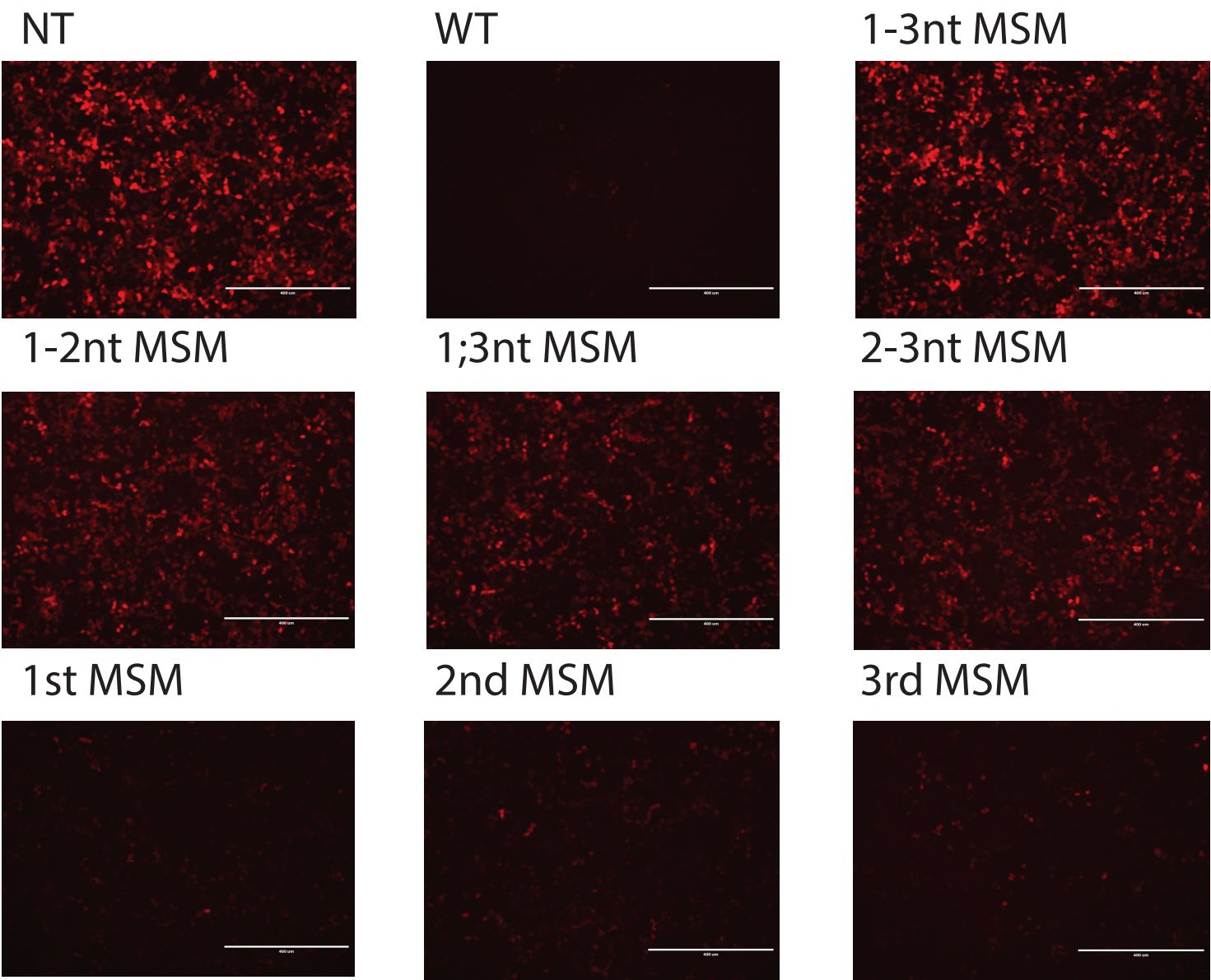

Supplement: Supplementary file 7 — Source data for Extended Data Fig. 5. [file 41594_2024_1336_MOESM7_ESM.pdf]

Figure 4k

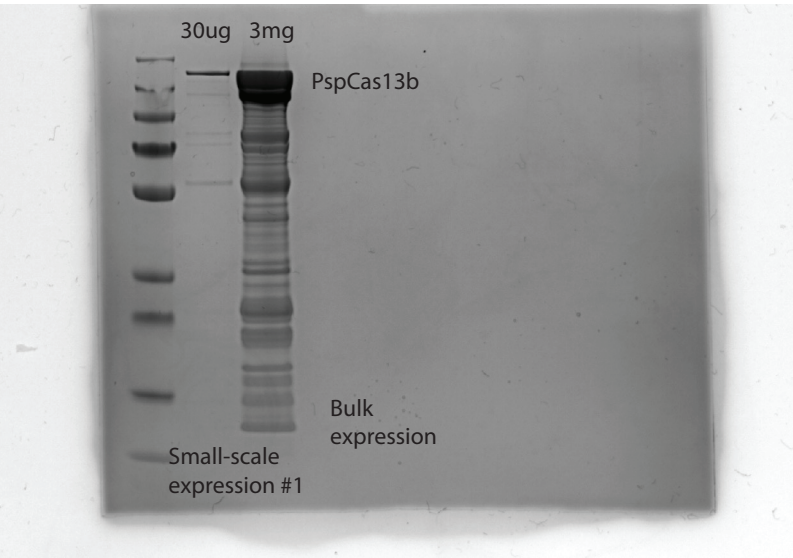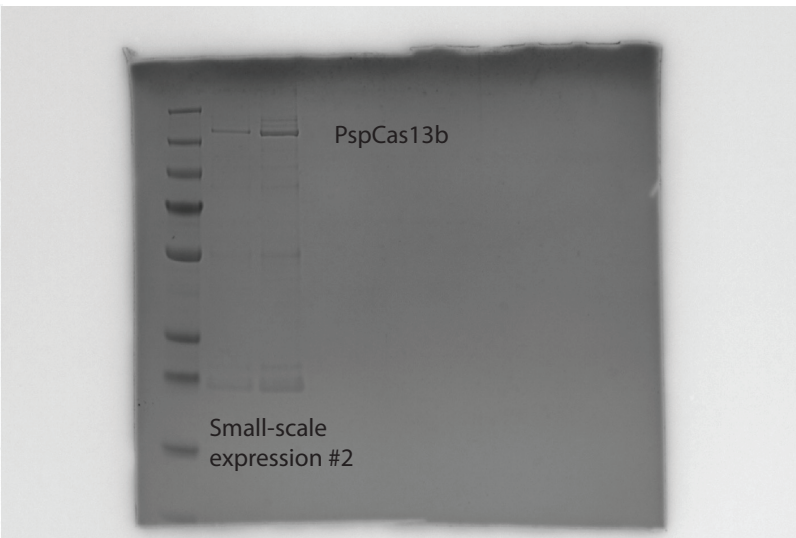

Figure 4l

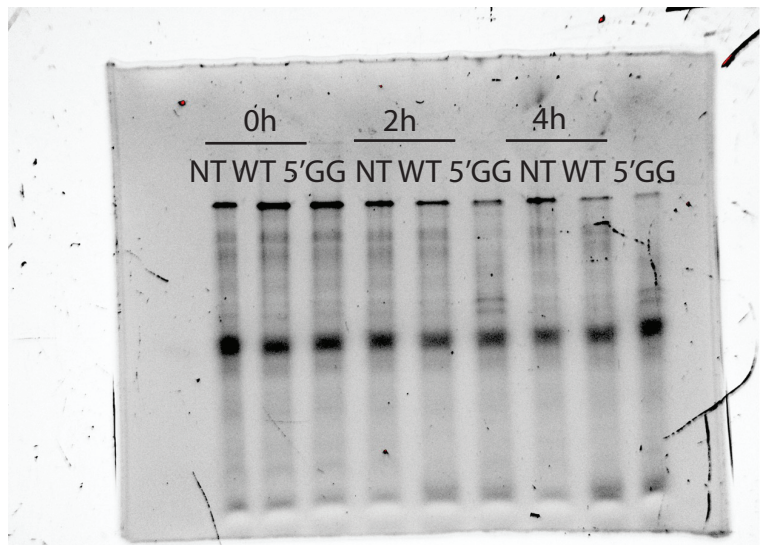

Figure 4m

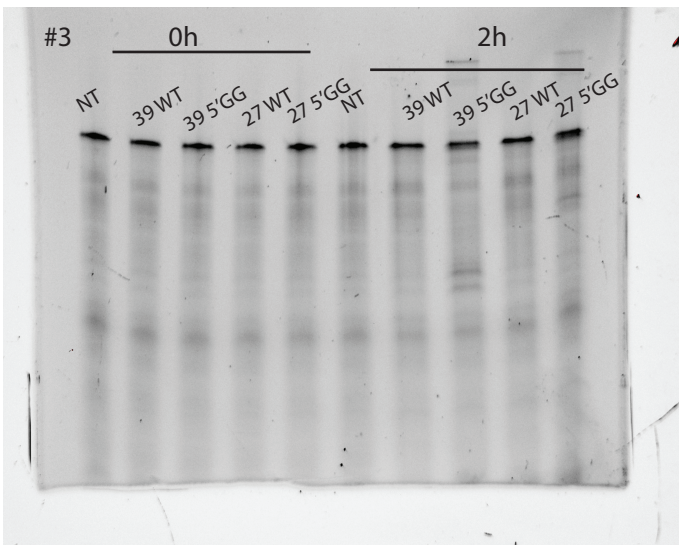

Figure 4m

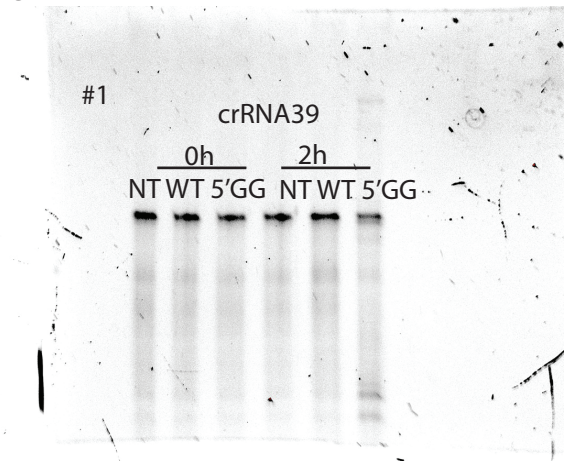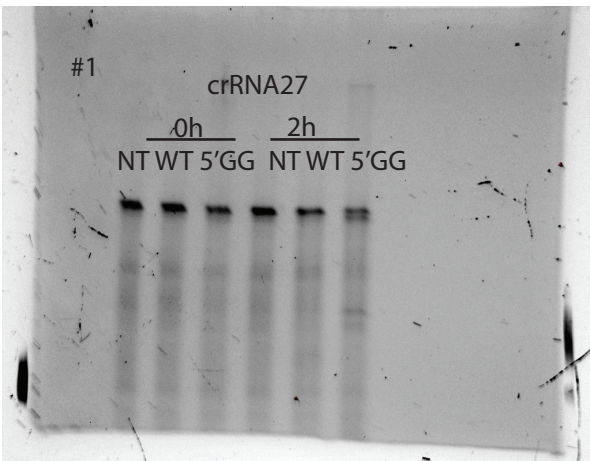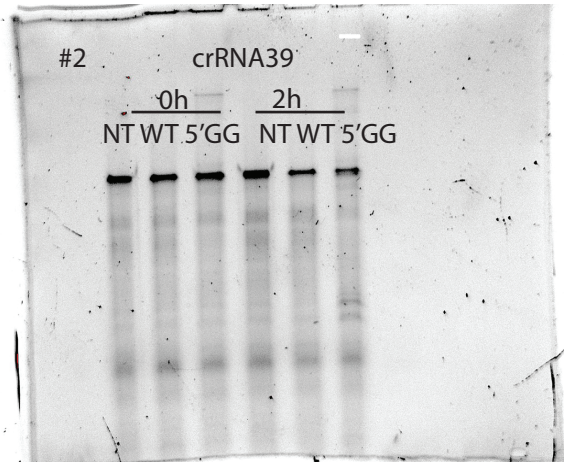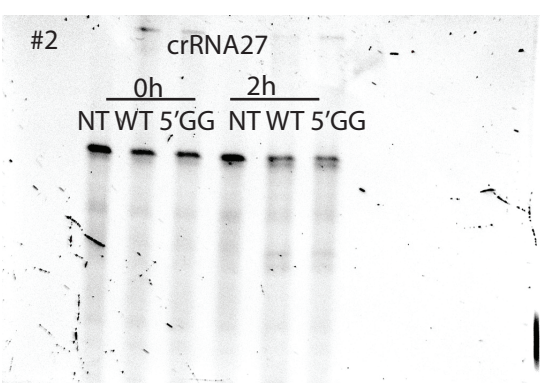

Supplement: Supplementary file 13 — Unprocessed western blots and gels. [file 41594_2024_1336_MOESM13_ESM.pdf]

Figure 7d

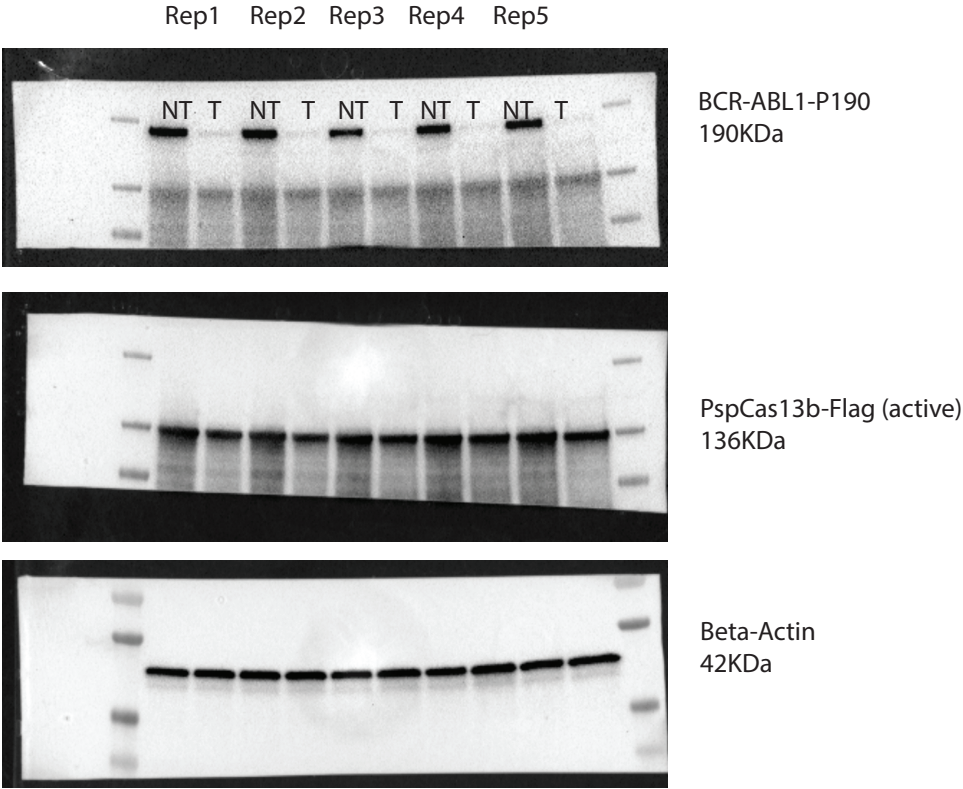

Figure 7h

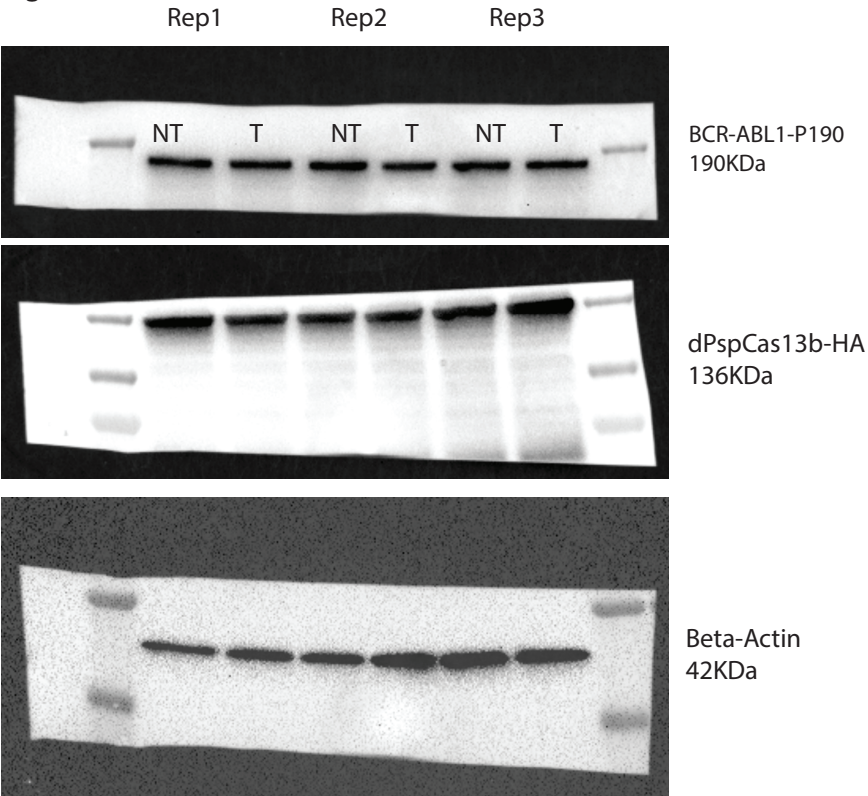

Figure 7l

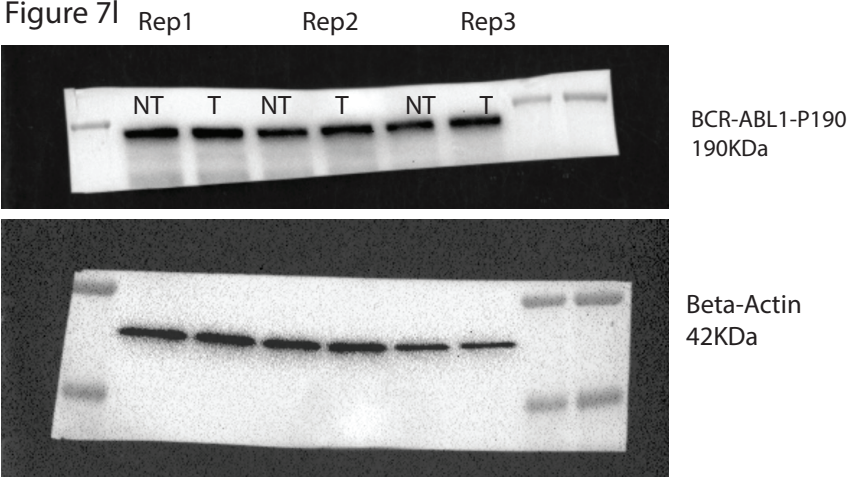

Supplement: Supplementary file 17 — Unprocessed western blots. [file 41594_2024_1336_MOESM17_ESM.pdf]

Extended Data Figure 6f

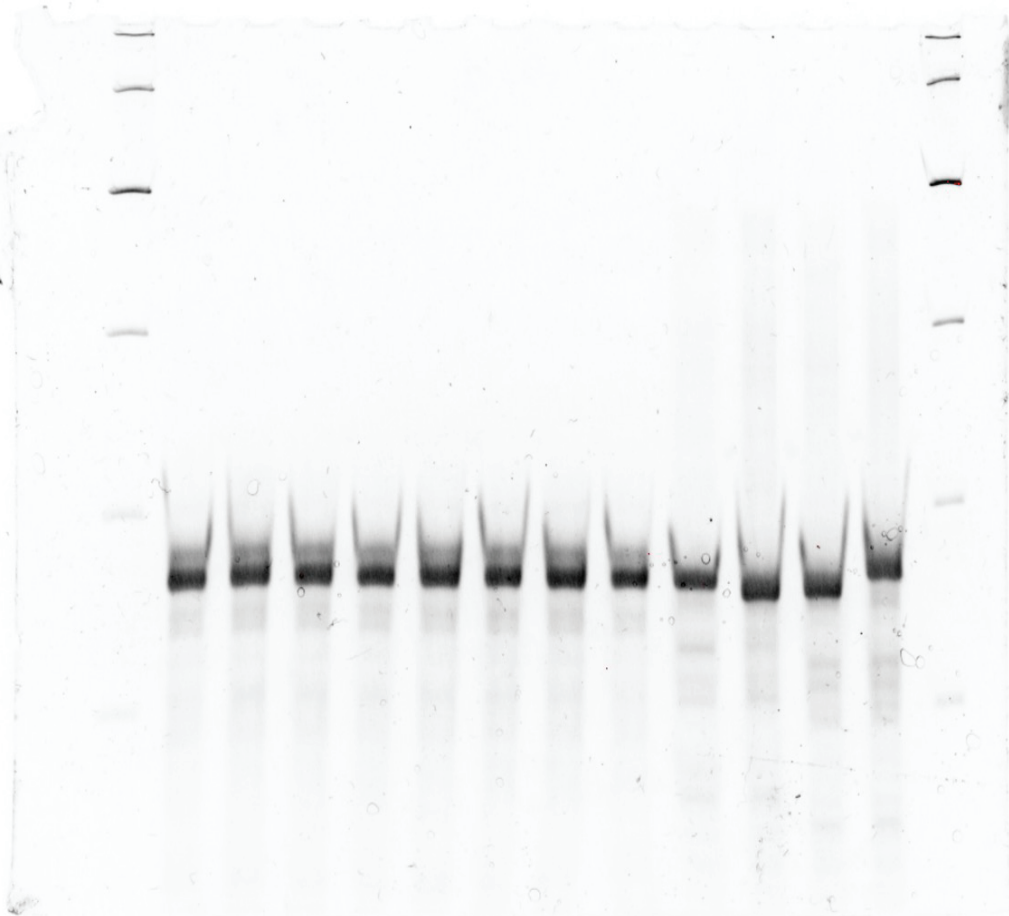

Extended Data Figure 6g

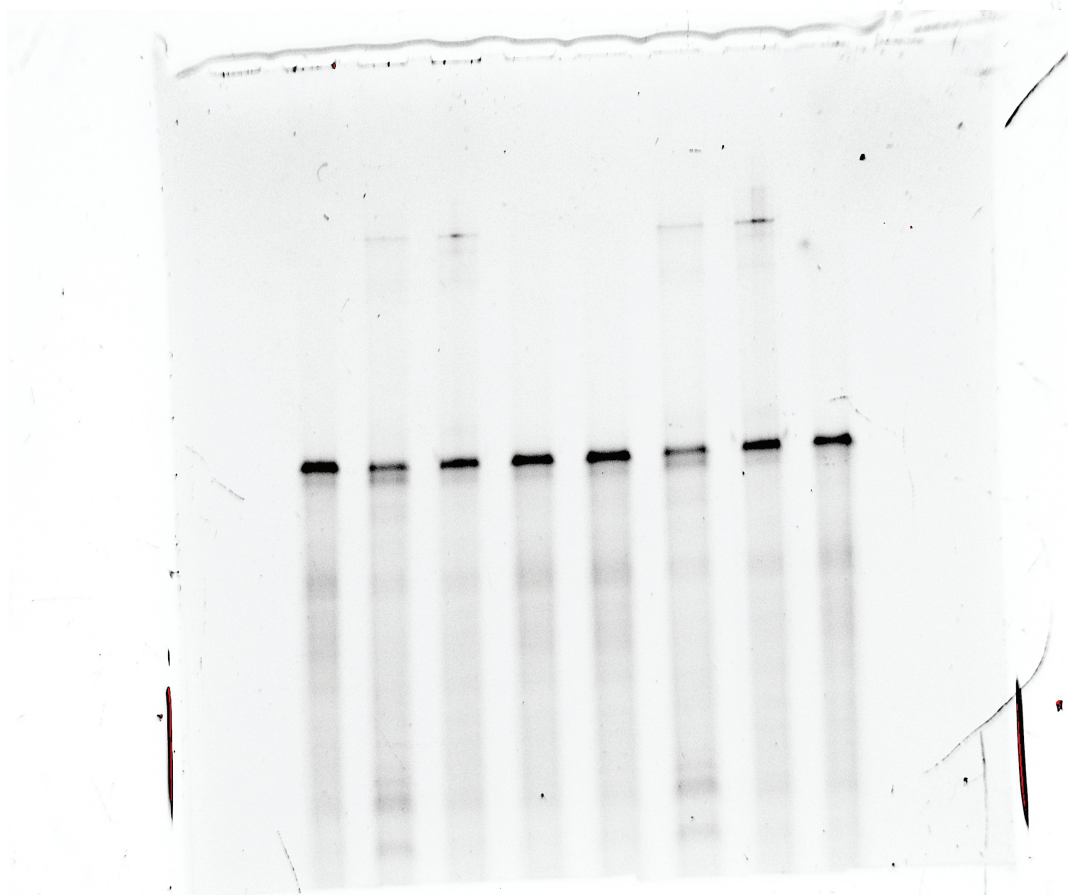

Supplement: Supplementary file 23 — Unprocessed gels. [file 41594_2024_1336_MOESM23_ESM.pdf]

crRNA39 5'GG

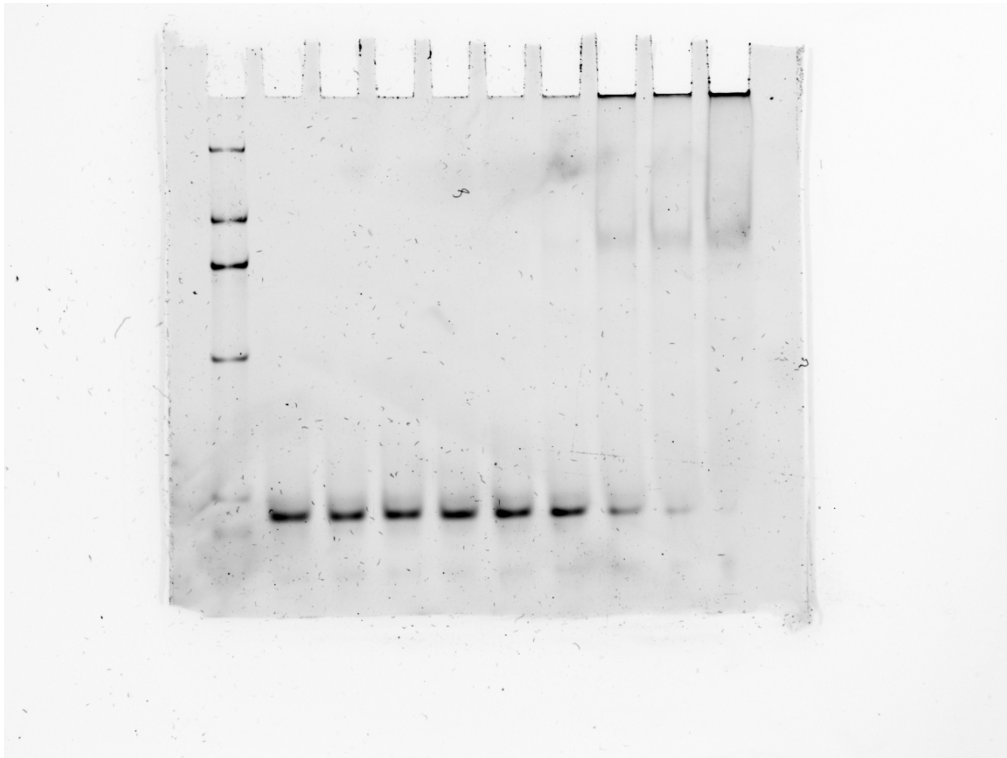

crRNA39 WT

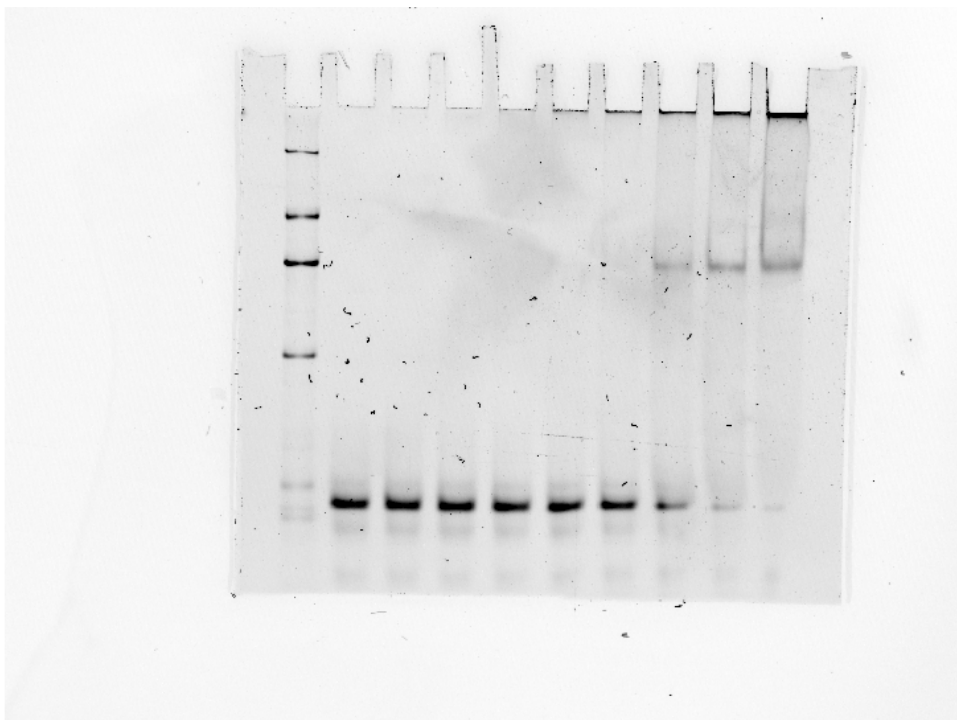

Supplement: Supplementary file 24 — Unprocessed gels. [file 41594_2024_1336_MOESM24_ESM.pdf]
