# Supplementary material for: Single-base tiled screen unveils design principles of PspCas13b for potent and off-target-free RNA silencing
Source: Nat Struct Mol Biol. 2024 Jul 1;31(11):1702–16. doi: 10.1038/s41594-024-01336-0 (PMC11564092; doi:10.1038/s41594-024-01336-0)
Supplement: Supplementary file 9 — Unprocessed western blots. [file 41594_2024_1336_MOESM9_ESM.pdf]

Figure 1g

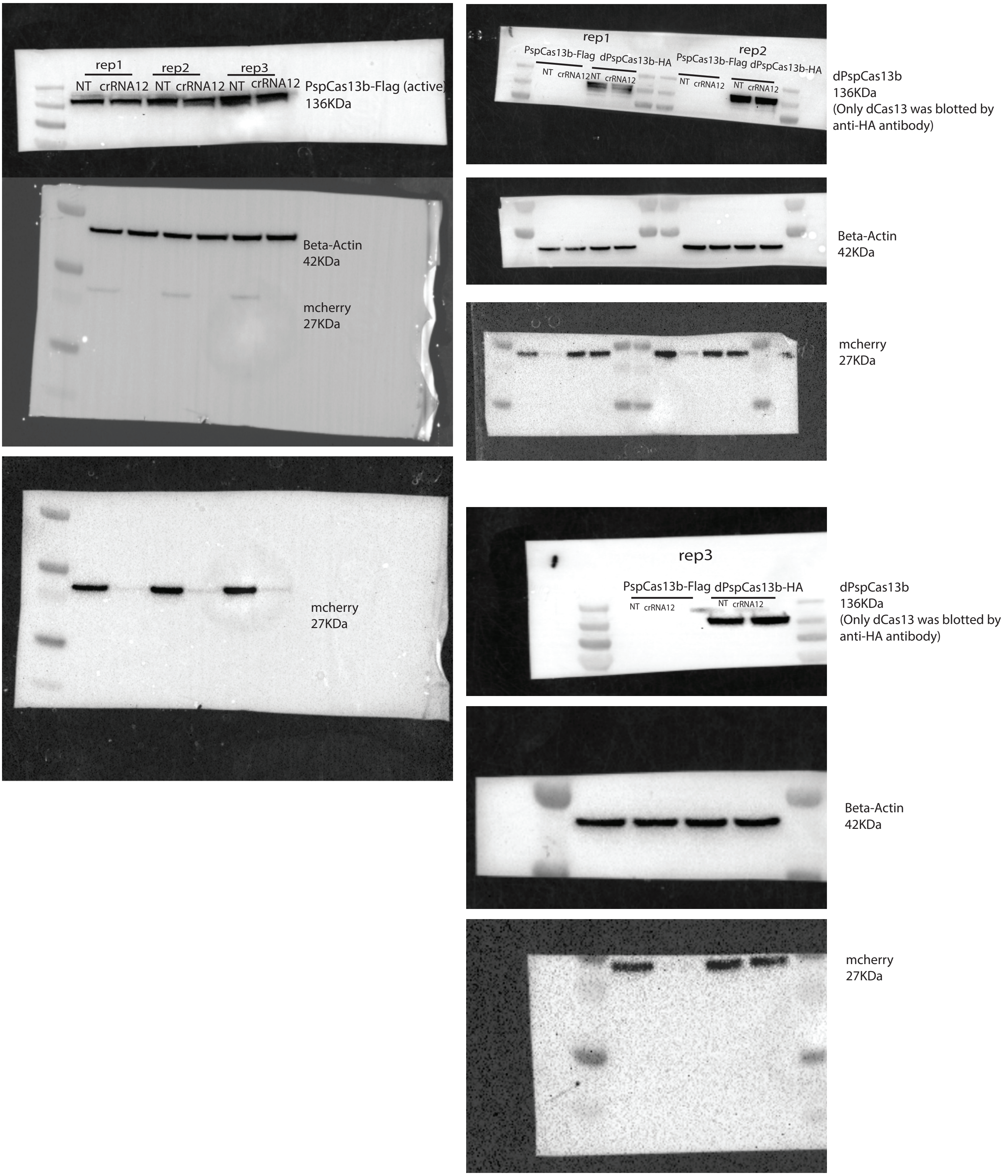

rep1
rep2
rep3
NT crRNA12 NT crRNA12 NT crRNA12
PspCas13b-Flag (active)
136KDa
Beta-Actin
42KDa
mcherry
27KDa
mcherry
27KDa
rep1
rep2
PspCas13b-Flag dPspCas13b-HA PspCas13b-Flag dPspCas13b-HA
NT crRNA12 NT crRNA12 NT crRNA12 NT crRNA12
dPspCas13b
136KDa
(Only dCas13 was blotted by
anti-HA antibody)
Beta-Actin
42KDa
mcherry
27KDa
rep3
PspCas13b-Flag dPspCas13b-HA
NT crRNA12 NT crRNA12
dPspCas13b
136KDa
(Only dCas13 was blotted by
anti-HA antibody)
Beta-Actin
42KDa
mcherry
27KDa
